# European Hedgehogs (*Erinaceus europaeus* L.) as a Reservoir of Dermatophytes in Poland

**DOI:** 10.1007/s00248-021-01866-w

**Published:** 2021-09-18

**Authors:** Sebastian Gnat, Dominik Łagowski, Mariusz Dyląg, Aneta Nowakiewicz

**Affiliations:** 1 Department of Veterinary Microbiology, Faculty of Veterinary Medicine, University of Life Sciences, Akademicka 12, 20-033 Lublin, Poland; 2 Department of Mycology and Genetics, Faculty of Biological Sciences, University of Wroclaw, Wroclaw, Poland

**Keywords:** *Erinaceus europaeus*, Dermatophyte reservoir, Prevalence, Wildlife, Urban areas, Zoonoses

## Abstract

The European hedgehog (*Erinaceus europaeus* Linnaeus) frequently colonises areas located close to human life in cities, as these are more suitable nest sites offering an abundance of food and allowing avoidance of predators. However, urbanisation has a significant impact on the epidemiology of infectious diseases, including dermatophytoses, the primary source of which are wild animals. In this study, we determined the spectrum of dermatophytes isolated from the European hedgehog and assessed their susceptibility profile to antifungal drugs. Symptomatic and asymptomatic dermatophyte infections were observed in 7.7% and 8% of the 182 examined free-living hedgehogs, respectively. In the pool of the isolated dermatophyte strains, *Trichophyton erinacei* was dominant (29.9%), followed by *Trichophyton mentagrophytes* (17.9%), *Trichophyton benhamiae* (13.4%), *Nannizzia gypsea* (11.9%), *Microsporum canis* (10.4%), *Nannizzia nana* (7.5%), *Paraphyton cookei* (6.0%), and *Nannizzia fulva* (3.0%). Susceptibility tests revealed the highest activity of luliconazole and the lowest of activity fluconazole among the azole drugs applied. Although terbinafine generally exhibited high efficacy, two *Trichophyton mentagrophytes* isolates showed resistance to this drug (MIC = 2 µg/ml) resulting from missense mutations in the SQLE gene corresponding to the amino acid substitution Leu393Phe. Summarising, our study has also revealed that such wildlife animals as hedgehogs can be a reservoir of pathogenic human dermatophytes, including harmful strains resistant to commonly used antifungal drugs.

**Graphical Abstract:**

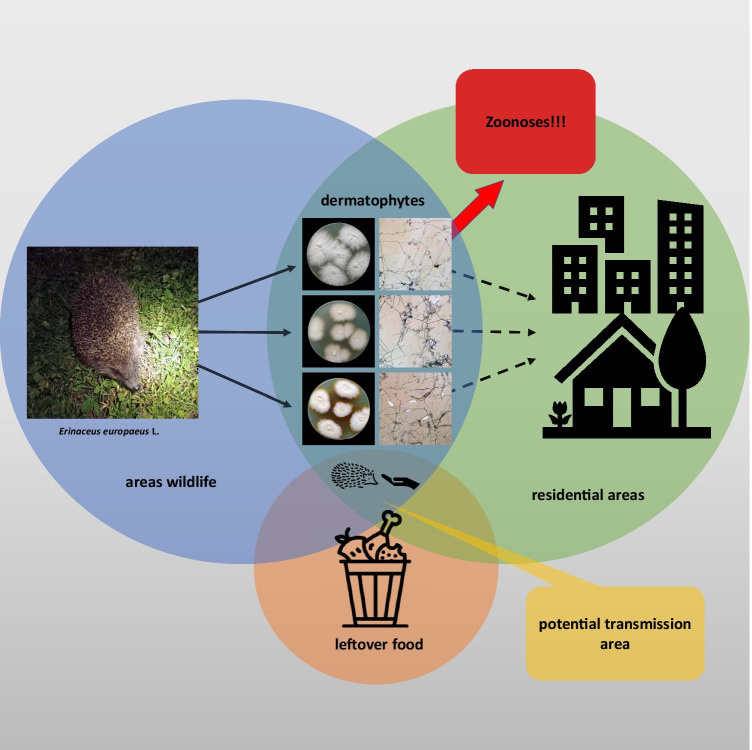

## Introduction

In the last few years, superficial infections caused by filamentous fungi, especially dermatophytes, along with the concomitant increase in the number of difficult-to-treat cases have increasingly been recognised worldwide as a serious public health problem [1–3]. This imposes a high economic burden, as approximately $1.67 billion is spent on the treatment of dermatophytosis each year [4]. The main etiological factors of superficial mycoses are dermatophytes, which are a cosmopolitan group encompassing more than 50 species classified within the genera *Trichophyton*, *Microsporum*, *Epidermophyton*, *Arthroderma*, *Nannizzia*, *Lophophyton, Guarromyces,* and *Paraphyton* [5]. The sources of dermatophytes include the natural environment, i.e. soil (geophilic species), and transmission via direct or indirect contact with infected humans (antropophilic species) or animals (zoophilic species) as well as asymptomatic carriers [6–8].

The widely reported factors that predispose to human dermatophytosis include improper hygiene, occlusive footwear, socioeconomic conditions, profession, animal breeding, diabetes mellitus, age, genetics, skin maceration, and immunocompromised status [7, 9, 10]. Moreover, contact with domestic and wild animals should also be considered a risk factor [11, 12]. The changing natural environment is favourable for these animals to colonise sites located close to human life in cities. It is commonly known that urbanisation has a significant impact on the epidemiology of infectious diseases, including dermatophytoses [13]. Many interventions, also nonprofessional in wildlife rescue, raise questions concerning the risk of transmission of zoophilic dermatophytes to volunteers. Additionally, the prevalence of symptomatic infections and the carriage of dermatophytes in wild animals have not yet been defined in detail.

Adaptation to urban and suburban areas is characteristic of the populations of the European hedgehog (*Erinaceus europaeus* Linnaeus, 1758), also known as the West European hedgehog or the common hedgehog [14, 15]. In the 80 s and 90 s of the last century, a decline of the hedgehog population was described in western and central European regions, which was reflected in the inclusion of the species in Appendix III of the Bern Convention on the Conservation of European Wildlife and Natural Habitats [16]. The potential cause of this phenomenon may be the aftermath of the loss of their natural habitat and food sources after urbanisation, agriculture intensification, and the use of pesticides [17]. Other studies indicate that hedgehogs may prefer urban areas, as they offer more suitable nest sites and ensure a lower risk of predation from European badgers (*Meles meles*) [18]. Although hedgehogs residing in urban areas primarily become active after midnight and avoid foraging near roads, which is likely to reduce the dangers and disturbances caused by human activities such as vehicle and foot traffic and the disturbances from dogs, their contact with humans and pets is reported more frequently every year [19, 20]. The major microbial infections associated with hedgehogs have bacterial aetiology, i.e. *Salmonella* spp. and *Mycobacterium* spp. [21, 22]. Furthermore, it is commonly known that European hedgehogs can also constitute an essential reservoir of dermatophytes, especially *Trichophyton erinacei* [12, 16]. Nevertheless, the available information about the prevalence of dermatophytosis and carriage status in hedgehogs is based on a small dataset of free-ranging healthy hedgehogs or on a larger number of sick-injured hedgehogs in wildlife rescue centres. Hence, there seems to be a necessity to control the prevalence of dermatophytes in hedgehogs and to establish health precautions to avoid zoonotic infections. The latter may also apply to children who have contact with soil or pets encountering hedgehogs or their excrements, including keratinised tissue fragments, on the property of their owners.

**Fig. 1:**
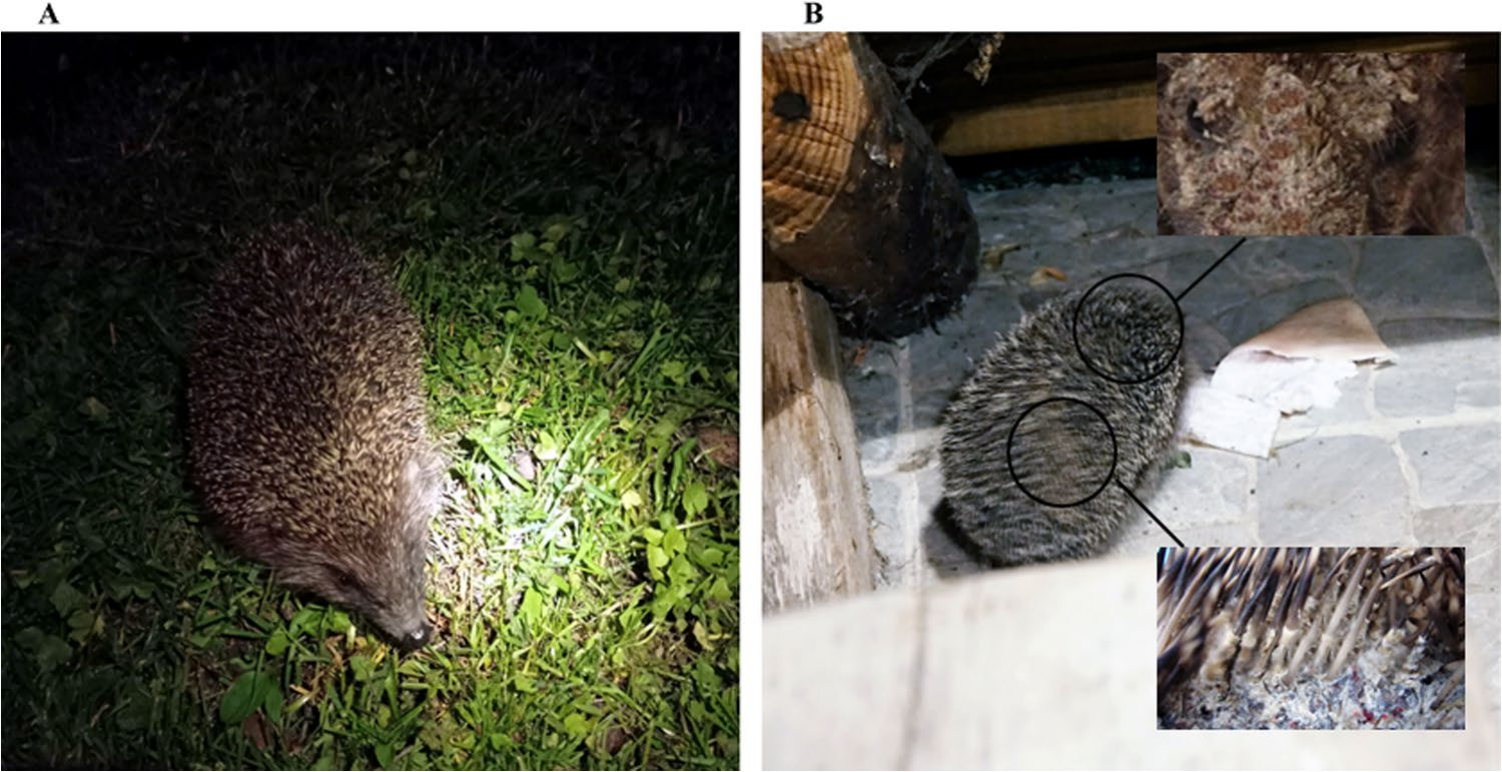
Free-living hedgehog (*Erinaceus europaeus* Linnaeus) during clinical examination in urbanised areas. Asymptomatic animal (**A**) and hedgehog with multiple lesions (**B**)

The aim of this study was to determine the prevalence of dermatophytes on the body integuments of European hedgehogs in Poland. Comparative analyses were carried out over a 5-year period. In addition, species of dermatophytes were identified using conventional and molecular methods to indicate dermatophyte species associated with hedgehogs. Finally, antifungal drug susceptibility tests were performed due to the common phenomenon of antifungal drug resistance among strains of zoophilic dermatophytes [23–25], whose growing number is a potential risk for public health (Fig. 1).

## Materials and Methods

### Material Sampling from Hedgehogs

In total, 182 samples were collected from asymptomatic hedgehogs (*n* = 148) and those with dermatophytosis (*n* = 34) from six localities in different regions in Poland during the period from May to August in 2016–2020 (Table 1). Each location was a city with more than 100,000 inhabitants located in the eastern, central, and southwestern parts of Poland. All animals included in the study were free-living specimens in urban areas. The field identification of *Erinaceus europaeus* L. was based on exterior characteristics, i.e. fur colouration and spine striping, presence or absence of a mask on the muzzle, and presence and shape of a white spot on the ventral side. Dermatophytosis was considered symptomatic when such skin lesions as spike loss, crusty skin, and erythema were diagnosed according to the criterion proposed by Bexton and Nelson [26]. Clinical examination was performed at the hedgehog encounter site without transferring the animal or any additional medicinal procedures in accordance with good veterinary practice standards. In the case of hedgehogs with symptomatic dermatophytosis, skin scrapings were collected by rubbing off a spike using a sterile scalpel and placed into a falcon tube. The asymptomatic animals were sampled by scrubbing the whole skin surface using the brush technique [27].

**Table 1:** Numerical data on the number of tested hedgehogs and the results obtained

Year	Number of samples			Number of isolates (%)
	Asymptomatic	Symptomatic	Total	
2016	28	6	34	13 (38.2)
2017	35	5	40	10 (25.6)
2018	26	15	41	19 (46.3)
2019	21	3	24	9 (39.1)
2020	38	5	43	14 (32.5)
Summarized	148	34	182	67 (36.8)

### Detection and Identification of Dermatophytes

**Fig. 2:**
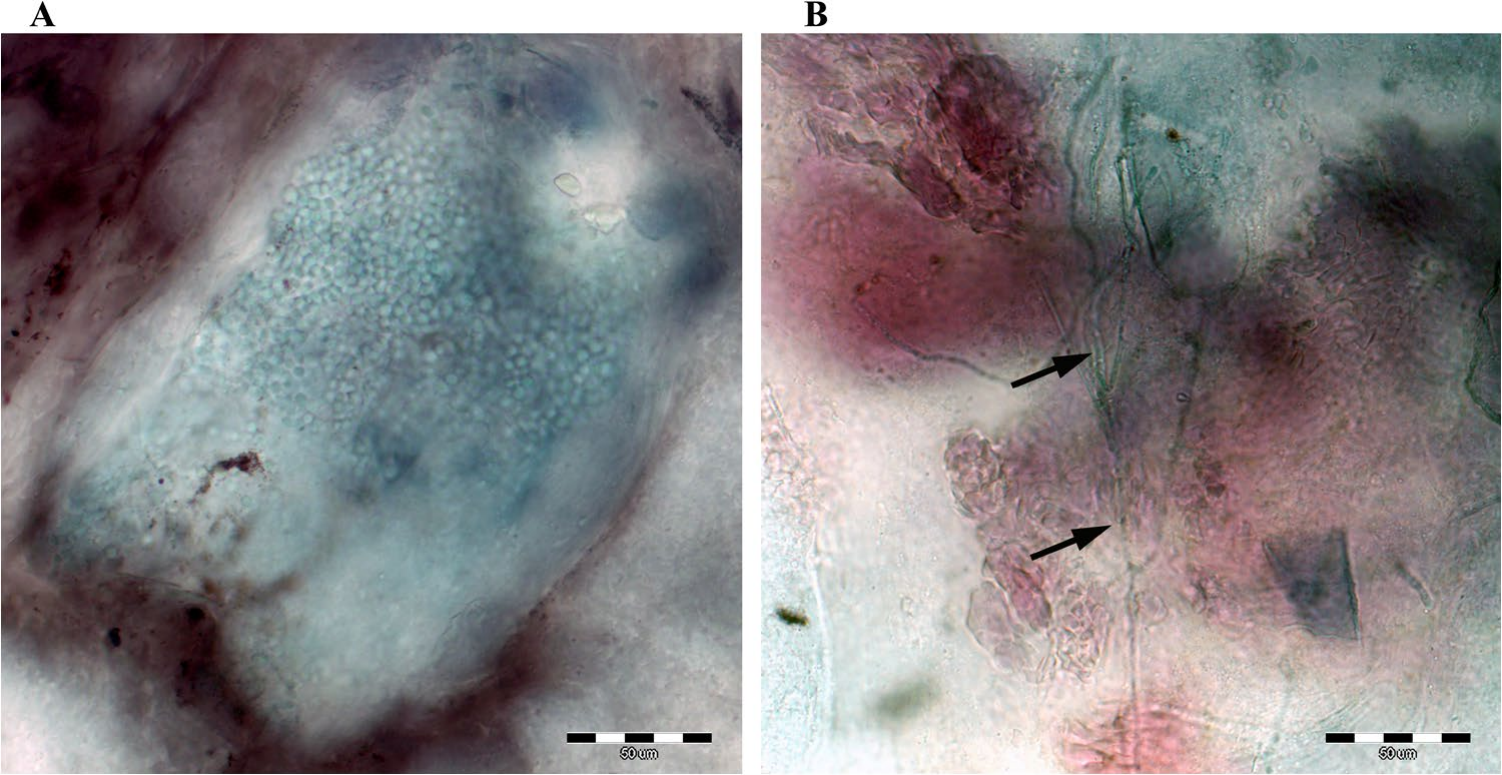
Direct preparation from hedgehog skin scrapings stained with chlorazol black E magnified 400 × (Olympus BX51, Tokyo, Japan). Notes: **A**, arthrospores in the skin scrap; **B**, mycelium fragments indicated by arrows

Mycological procedures, i.e. detection and species identification of the isolates, were performed with a few modifications as described previously by Gnat et al. [28]. The qPCR and microscopic methods were used to detect dermatophytes in the samples. Briefly, for the qPCR technique, DNA was extracted using a DNeasy Blood & Tissue Kit (QIAGEN, Hilden, Germany) according to the manufacturer’s instructions. The final positive result was obtained when the reactions with the pan‐dermatophyte primers (primer F: AGCGCYCGCCGRAGGA, primer R: GATTCACGGAATTCTGCAATTCAC) were positive. The real‐time PCR was performed in a final volume of 25 μl containing 12.5 μl 2 × of QuantiTect SYBR Green PCR Master Mix, 0.5 μl (20 pmol) of both forward and reverse primers, 3 μl of DNA, and 8.5 μl of water. The cycling conditions of the reaction performed using Stratagene Mx3005P (Agilent Technologies, Santa Clara, USA) were as follows: primary denaturation for 3 min at 96 °C, 45 denaturation cycles for 10 s at 96 °C, annealing for 1 min at 65 °C, and elongation for 30 s at 72 °C. The melting curve analysis was performed in the following conditions: 1 min at 94 °C, 1 min at 65 °C, and 1 min at 94 °C. Direct examination of the skin scrapings was carried out using light and fluorescence microscopes after suspending the samples in clearing fluid comprising 10% KOH in a DMSO (dimethyl sulfoxide) solution. Microscopic preparations were then examined under a magnification of 400 × (Olympus BX51, Tokyo, Japan). (Fig. 2) For light microscopy, the preparations were examined after lactophenol blue and chlorazol black (Sigma-Aldrich, St. Louis, MO, USA) staining. Calcofluor white (Sigma-Aldrich, St. Louis, MO, USA) staining was used for fluorescence microscopy with 300–440 nm emission and ca. 355 nm excitation wavelengths.

**Fig. 3:**
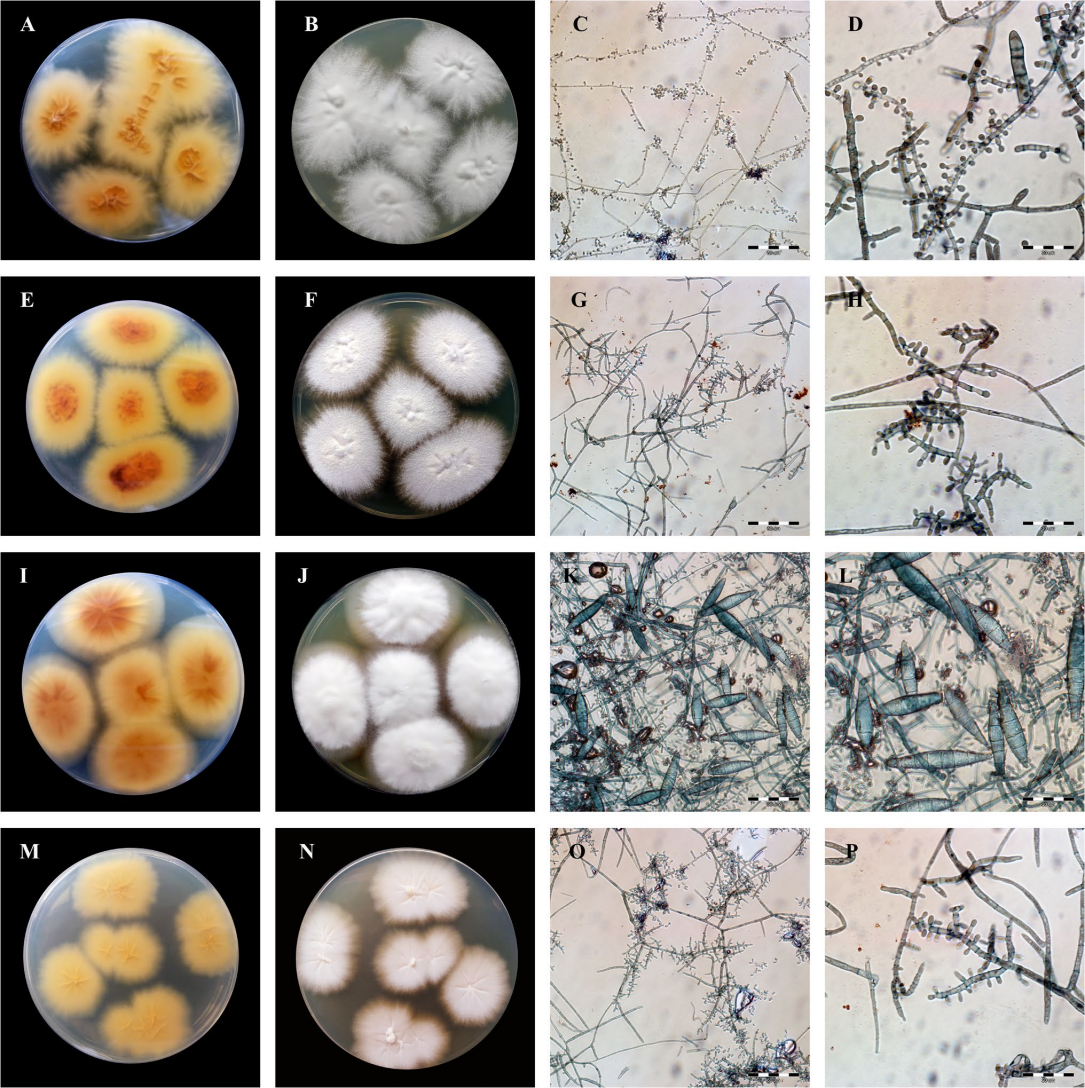

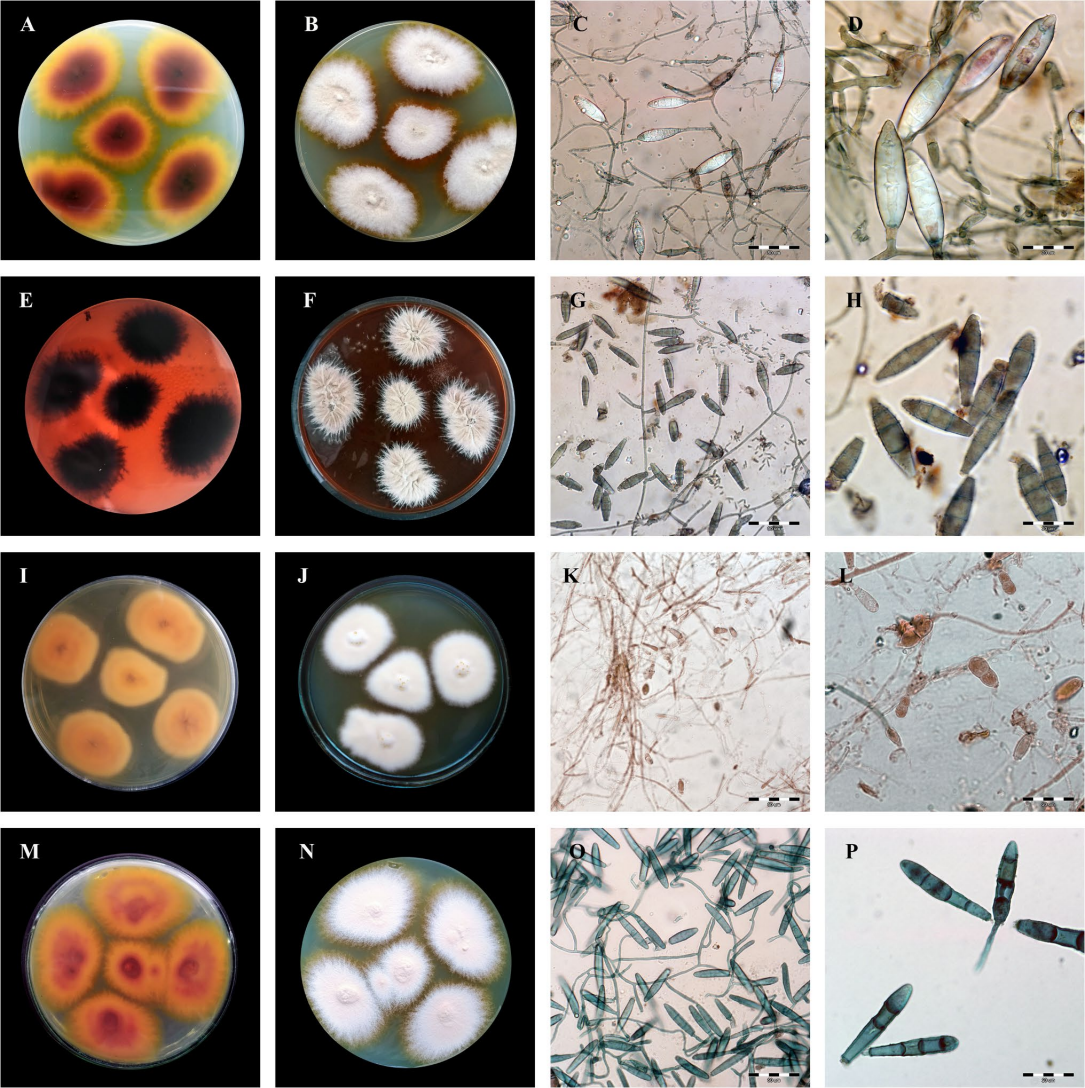
Macro- and micromorphology of dermatophytes isolated from free-living hedgehogs. Notes: fungi cultured on Sabouraud medium at 28 °C for 21 days. **A**–**D**, *Trichophyton mentagrophytes*; **E**–**H**, *Trichophyton benhamiae*; **I**–**L**, *Microsporum canis*; **M**–**P**, *Trichophyton erinacei.* **A**, **E**, **I**, and **M**, obverse appearance; **B**, **F**, **J**, and **N**, reverse appearance; **C**, **G**, **K**, and **O**, micromorphology after staining with chlorazol black E at 400 × magnification (Olympus BX51, Tokyo, Japan); **D**, **H**, **L**, and **P**, micromorphology after staining with chlorazol black E at 1000 × magnification (Olympus BX51, Tokyo, Japan). **3B** Macro- and micromorphology of dermatophytes isolated from free-living hedgehogs continued. Notes: fungi cultured on Sabouraud medium at 28 °C for 21 days. **A**–**D**, *Nannizzia gypsea*; **E**–**H**, *Paraphyton cookei*; **I**–**L**, *Nannizzia nana*; **M**–**P**, *Nannizzia fulva*; **A**, **E**, **I,** and **M**, obverse appearance; **B**, **F**, **J**, and **N**, reverse appearance; **C**, **G**, **K**, and **O**, micromorphology after staining with chlorazol black E at 400 × magnification (Olympus BX51, Tokyo, Japan); **D**, **H**, **L**, and **P**, micromorphology after staining with chlorazol black E at 1000 × magnification (Olympus BX51, Tokyo, Japan)

Species identification was based on macro- and micro-morphological examinations of the obtained cultures and ITS (internal transcribed spacer) region sequencing and analysis. (Fig. 3A and B) Cultures were inoculated onto Sabouraud glucose agar containing 0.005% chloramphenicol and 0.05% cycloheximide (BioMaxima, Lublin, Poland) and on dermatophyte test medium (BioMaxima, Lublin, Poland) at 30 °C for 21 days. Additionally, DNA was isolated from the axenic culture with the phenol–chloroform method described by Gnat et al. [29]. Molecular identification was performed by amplification of the internal transcribed spacer (ITS) region and nucleotide sequence analysis using the ITS1 (5′-TCCGTAGGTGAACCTGCGG-3′) and ITS4 (5′-TCCTCCGCTTATTGATATGC-3′; Genomed, Warsaw, Poland) primer pair [30]. The PCR reaction conditions were as follows: initial step at 95 °C for 3 min followed by 30 cycles at 95 °C for 1 min, 50 °C for 1 min, and 72 °C for 1 min, and then a final extension step at 72 °C for 10 min. Electrophoretic separation of PCR products was carried out in 2% agarose gels. The ITS sequencing reaction was carried out using a BigDye Terminator Cycle Sequencing Kit (Life Technologies, Carlsbad, CA, USA). Two separate reactions were carried out using either the ITS1 or ITS4 primer. The PCR product was purified using an ExTerminator kit (A&A Biotechnology, Gdynia, Poland), and then the DNA sequence was read in a 3500 Genetic Analyser (Life Technologies, Carlsbad, CA, USA). All obtained nucleotide sequences were deposited in GenBank (Table 1).

### Susceptibility Testing to Antifungal Drugs

In vitro susceptibility of isolated dermatophyte strains towards allylamine, polyene, imidazole, triazole, and pyridinone derivative drugs was determined according to Clinical and Laboratory Standards Institute (CLSI) document M38Ed3 [31]. Reagent-grade amphotericin B (AMB), ciclopirox (CPO), griseofulvin (GRE), itraconazole (ITR), ketoconazole (KTC), miconazole (MCZ), naftifine (NFT), terbinafine (TRB), and voriconazole (VRC) were obtained in the powder form. Drug stock solutions were prepared in dimethyl sulfoxide (DMSO) to reach the final DMSO concentration in the wells below 1%. The drugs were analysed in two-fold serial dilutions in the range of 0.001–32 μg/ml. The dermatophyte isolates were cultured on potato dextrose agar (PDA; Difco) for 21 days, and suspensions comprising mostly conidia were prepared by gently scraping mature colonies into sterile physiological saline containing 0.002% Tween 80. Homogeneous inoculum supernatants were collected, their optical density (OD) at 530 nm was adjusted spectrophotometrically to transmission ranging from 65 to 70%, and the final density of the inoculum was from 1 × 10^3^ to 3 × 10^3^ CFU/ml. The inocula were diluted 1:50 in RPMI 1640 medium and incubated with the indicated concentrations of the antifungals in 96-well plates at 30 °C for 72 h. Minimum inhibitory concentrations (MICs) were read spectrophotometrically using a Varioskan LUX multimode microplate reader (Thermo Fisher Scientific) at 530 nm wavelength (*λ*). The endpoint for the minimal inhibitory concentration (MIC) was the antifungal concentration at which prominent inhibition of growth, i.e. ≥ 80% of that of the control was observed. *Trichophyton interdigitale* ATCC4439 and *T. rubrum* ATCC4438 served as quality controls for every new series of susceptibility tests. All the tests were performed in triplicate and each tested compound used in this study was purchased from Sigma-Aldrich (MO, USA) if not stated otherwise. In addition, the selection of the terbinafine-resistant *T. mentagrophytes* isolates was based on fungal growth on Sabouraud glucose agar (SGA, BioMaxima, Lublin, Poland) containing 1 µg/ml of this substance, and the growth rate was compared to the control (SGA without drug) after 7, 10, and 14 days.

### Squalene Epoxidase (SQLE) Gene Analysis for Terbinafine-Resistant T. mentagrophytes

Partial squalene epoxidase gene sequences for terbinafine-resistant isolates were analysed as previously described by Yamada et al. [32] with a few modifications. In short, the SQLE gene amplification reaction was carried out with primer pairs TrSQLE-F1 (5′-ATGGTTGTAGAGGCTCCTCCC-3′) and TrSQLE-R1 (5′-CTAGCTTTGAAGTTCGGCAAA-3′) and DNA template with a concentration of 50 ng/µl. The PCR reaction conditions were as follows: 30 cycles consisting of denaturation (1 min, at 95 °C), primer annealing (30 s, at 55 °C), and elongation (3 min, at 72 °C). The PCR products were separated on 2% agarose gel stained with ethidium bromide and visualised in UV light. The SQLE gene sequencing reaction was carried out using a BigDye Terminator Cycle Sequencing Kit (Life Technologies, Carlsbad, CA, USA) and one of the primers TrSQLE-F1 or TrSQLE-R1 purified using an ExTerminator kit (A&A Biotechnology, Gdynia, Poland). Next, the DNA sequence was read in a 3500 Genetic Analyser purchased from Life Technologies (Carlsbad, CA, USA). The nucleotide and predicted amino acid sequences obtained in MEGA ver. 7.0 software (https://www.megasoftware.net/) of the SQLE gene in the case of all the *T. mentagrophytes* isolates were compared with the reference sequences available in the GenBank database.

## Results

In total, 182 free-living hedgehogs were examined in this study. The veterinary analysis performed during the collection of the material showed 98 adult animals (53.9%), 65 juveniles (35.7%), and 19 hoglets (10.4%). Moreover, the sex rate was not homogeneous for the three age groups, and 109 hedgehogs were male (59.9%) and 73 animals were female (40.1%). During the field examination of hedgehogs, the veterinarian observed fleas (*Archaeopsylla erinacei*) on 42 animals (23.1%) and ticks (*Ixodes* sp.) on 21 animals (11.5%) or both ectoparasites on 34 animals (18.7%), with variable degrees of infestation. Thirty-one out of the 182 hedgehogs (17%) had skin lesions diagnosed as cutaneous myiasis. *Calliphoridae* (Diptera) eggs and/or larvae were detected in these animals. Additionally, in 20 hedgehogs with cutaneous myiasis, there were 12 animals (6.6%) with symptomatic dermatophytosis and 8 specimens (4.4%) were asymptomatic carriers.

The qPCR technique facilitated the detection of the genetic material of dermatophytes in 100% of samples taken from the symptomatic hedgehogs (34/34). Moreover, this method used to analyse the material from the asymptomatic carrier animals revealed that 45.3% (67/148) of the samples contained dermatophyte genetic material (Table 2). In turn, the direct light and fluorescence microscopy examination of the material sampled from the skin lesions in the hedgehogs revealed the presence of arthrospores in all samples. During the diagnostics of the skin scrapings from the asymptomatic animals, fungal elements were shown by the direct examination with light and fluorescence microscopy in 33.8% (50/148) and 37.8% (56/148) of the analysed cases, respectively. In turn, dermatophyte cultures were obtained from 36.8% hedgehogs (67/182), with 20 samples positive to *T. erinacei* (29.9%), 12 samples positive to *T. mentagrophytes* (17.9%), 9 samples positive to *T. benhamiae* (13.4%), and 26 samples positive to other species of dermatophytes (41.8%). All dermatophyte strains cultured in this study are presented in Table 3. The macro- and micro-morphological characteristics of the isolates classified within individual species were specific and allowed initial identification (Fig. 1). The main diagnostic criteria to distinguish between these species were the colony diameter and appearance, the presence of spiral hyphae, and species-specific macroconidia [33]. Furthermore, the same proportion of males (38, 56.7%) and females (29, 43.3%) were dermatophyte-positive in the culture tests. Similar proportions were also observed in dermatophyte culture-positive hedgehogs classified by age: 24 out of 67 were adults (35.8%), 22 were juveniles (32.8%), and 21 were hoglets (31.3%). Chi-square tests of independence showed no association between the detection of dermatophytes and the sex and age groups (*p* > 0.05). Additionally, 34 hedgehogs (50.7%) had clinical lesions indicative of dermatophytosis. There were only 12 out of the 34 symptomatic hedgehogs with cultures yielding 10 or more colonies per plate (35.3%), and no statistical association between the number of dermatophyte colonies and the presence of skin lesions was observed (*χ*^2^, *p* > 0.05). In turn, 20 of the symptomatic animals had wounds on the skin or were infested by *Calliphoridae* eggs and larvae (58.8%). The statistical analysis revealed a significant relationship between these variables, i.e. animals with skin wounds were more likely to suffer from dermatophytosis (*χ*2, *p* < 0.05). The head of the hedgehogs was observed as the most frequent site of infection, as 21 animals presented skin lesions in this body area (61.8%). Asymptomatic hedgehogs with isolated dermatophytes constituted 33 samples out of 148 subjects (22.3%). Cultures with less than ten colonies per plate were obtained from 28 of these positive animals (84.8%).

**Table 2:** Diagnostic effectiveness of qPCR, direct microscopy, and culture methods in relation to clinical material taken from hedgehogs

Type of infection	Method [% of positive results]				
	qPCR	Direct analysis		Cultures	
		In light microscopy	In fluorescence microscopy	Less than 10 colonies	More than 10 colonies
Symptomatic	100	100	100	64.7	35.3
Asymptomatic	45.3	33.8	37.8	18.9	3.4

**Table 3:** Dermatophyte species isolated from hedgehogs with the GenBank accession numbers of the nucleotide sequences

Dermatophyte species	Type of infection	Number of isolates		GenBank accession numbers of ITS region and SQLE gene nucleotide sequences
		No	Total (%)	
*Trichophyton erinacei*	Symptomatic	12	20 (29.9)	MW755314-MW755325
	Asymptomatic	8		MW755306-MW755313
*Trichophyton mentagrophytes*	Symptomatic	8	12 (17.9)	MW759405-MW759412 SQLE (2 strains): MZ065195-MZ065196
	Asymptomatic	4		MW759878-MW759881
*Trichophyton benhamiae*	Symptomatic	5	9 (13.4)	MW759866-MW759870
	Asymptomatic	4		MW759871-MW759874
*Microsporum canis*	Symptomatic	4	7 (10.4)	MW759862-MW759865
	Asymptomatic	3		MW759875-MW759877
*Nannizzia gypsea*	Symptomatic	3	8 (11.9)	MW759402-MW759404
	Asymptomatic	5		MW759413-MW759417
*Nannizzia nana*	Asymptomatic	5	5 (7.5)	MW755440-MW755444
*Nannizzia fulva*	Symptomatic	2	2 (3.0)	MZ052087-MZ052088
*Paraphyton cookei*	Asymptomatic	4	4 (6.0)	MW755326-MW755329

The MIC_50_ and MIC_90_ values of the thirteen antifungal drugs tested in the pool of 67 dermatophyte isolates obtained from the symptomatic and asymptomatic hedgehogs are summarised in Table 4. Luliconazole exhibited the lowest MIC_50_ and MIC_90_ values in comparison with the other drugs, regardless of the dermatophyte species. In turn, fluconazole was found to exert the weakest in vitro effect and had the highest MIC_50_ and MIC_90_ values. Although terbinafine exhibited high efficacy and low MIC_50_ values for all tested dermatophytes, two clinical isolates of *T. mentagrophytes* obtained from the symptomatic animals showed resistance to this substance (MIC = 2 µg/ml). The verification test was performed on Sabouraud’s medium supplemented with 1 μg/ml of terbinafine to confirm the resistance of these isolates to the drug. The partial sequences of the SQLE gene obtained for these terbinafine-resistant *T. mentagrophytes* strains (GenBank accession numbers: MZ065195 and MZ065196) harboured missense mutations corresponding to the amino acid substitution Leu393Phe.

**Table 4:** In vitro antifungal susceptibilities of dermatophyte isolates obtained from hedgehogs

Antifungal agents		MIC (µg/ml)	Dermatophyte species								
			*Trichophyton erinacei*	*Trichophyton mentagrophytes*		*Trichophyton benhamiae*	*Microsporum canis*	*Nannizzia gypsea*	*Nannizzia nana*	*Paraphyton cookei*	Nannizzia fulva*
Allylamine	NFT	MIC_50_	0.016	0.008		0.004	0.032	0.25	0.032	0.25	0.032
		MIC_90_	0.032	0.016		0.016	0.064	0.25	0.032	0.5	
	TRB	MIC_50_	0.004	0.008	(2)	0.008	0.016	0.0125	0.032	0.0125	0.032
		MIC_90_	0.0016	2		0.032	0.016	0.25	0.064	0.032	
Polyenes	AMB	MIC_50_	0.25	0.25		0.125	0.25	0.064	0.125	0.25	0.5
		MIC_90_	0.25	0.5		0.25	1	0.25	0.5	0.5	
Non-polyenes	GRE	MIC_50_	1	1		0.5	0.25	0.5	0.25	1	0.5
		MIC_90_	1	2		1	1	0.5	1	2	
Imidazoles	KTC	MIC_50_	0.5	0.5		0.25	0.125	0.125	1	0.5	0.5
		MIC_90_	0.5	2		0.5	0.5	0.125	1	1	
	MCZ	MIC_50_	0.032	0.064		0.016	0.032	0.064	0.5	0.125	0.064
		MIC_90_	0.125	0.125		0.125	0.064	0.125	2	0.25	
	ENC	MIC_50_	0.125	0.25		0.064	0.032	0.5	0.5	0.25	0.032
		MIC_90_	0.25	2		0.25	0.125	1	0.5	1	
	LUC	MIC_50_	0.004	0.004		0.016	0.008	0.016	0.008	0.016	0.008
		MIC_90_	0.016	0.008		0.032	0.016	0.064	0.064	0.016	
Triazoles	ITC	MIC_50_	0.25	0.5		0.125	0.125	0.25	0.125	0.25	0.25
		MIC_90_	1	2		0.25	1	0.5	1	1	
	FLC	MIC_50_	16	32		16	16	8	4	8	64
		MIC_90_	32	64		64	16	16	16	32	
	VRC	MIC_50_	0.032	0.016		0.032	0.064	0.016	0.125	0.25	0.125
		MIC_90_	0.125	0.064		0.25	0.125	0.064	0.5	1	
Pyridinone derivatives	CPO	MIC_50_	0.064	0.016		0.032	0.25	0.016	0.032	0.064	0.032
		MIC_90_	0.064	0.032		0.064	0.5	0.064	0.032	0.064	
Phenyl morpholine derivatives	AMR	MIC_50_	0.008	0.016		0.008	0.016	0.032	0.008	0.016	0.016
		MIC_90_	0.016	0.064		0.008	0.032	0.032	0.008	0.016	

Notes: abbreviations of antifungal substances: *AMB*, amphotericin B; *AMR*, amorolfine; *CPO*, ciclopirox; *ENC*, enilconazole; *FLC*, fluconazole; *GRE*, griseofulvin; *ITC*, itraconazole; *KTC*, ketoconazole; *LUC*, luliconazole; *MCZ*, miconazole; *NFT*, naftifine; *TRB*, terbinafine; *VRC*, voriconazole; (*n*), number of strains that showed resistance to terbinafine (MIC = 2 µg/ml); ^ST^statistically significantly the lowest result in human/animals group; *the result presented for the two obtained isolates

## Discussion

The European hedgehog (*Erinaceus europaeus* Linnaeus, 1758) is a nocturnal insectivorous mammal with a wide distribution throughout Europe [22, 34]. The hedgehog population has increased in urban areas, especially in cities [12, 17]. Although hedgehogs spend most of the daylight hours sleeping and emerge at night to forage, interactions between hedgehogs and other animals and humans are on the rise nowadays [22, 35]. This is confirmed by reports on contact urticaria in some handlers and a number of infectious zoonotic diseases associated with hedgehogs [21, 36, 37]. Several reports also demonstrate the ability of hedgehogs to transmit dermatophytes to humans, resulting in zoonotic disease [38, 39] [40]. Hence, monitoring of hedgehog populations in urban areas has become of utmost importance if we consider the role of wild animals as carriers of dermatophytes and related fungi.

In our study, the total rate of culture-positive samples from hedgehogs was 36.8%. This percentage is consistent with earlier results, although these studies were conducted in the 1960s. In 1964, Smith and Marples [41] described a higher prevalence of dermatophyte isolates (44.7%) within urban wild populations in New Zealand. In turn, in studies conducted in 1969 in Great Britain, Morris and English [42] found a lower percentage of dermatophytoses in hedgehogs (20–25%). In a more recent study conducted in wildlife rehabilitation centres in France, Le Barzic et al. [12] revealed a 25.4% rate of dermatophyte-positive samples from hedgehogs. Moreover, the same study showed 79.2% of positive cultures coming from asymptomatic hedgehogs [12]. This percentage is much higher than that obtained in our study. The asymptomatic dermatophyte-positive hedgehogs constituted only 22.3%, and cultures with less than ten colonies per plate were obtained in 84.8% of these samples. The differences between these results and those obtained in our study may be associated with the method of collecting samples. The wild animal rescue centre where skin scrapings were collected in the French studies houses sick, injured, or malnourished animals, in health status that may predispose them to dermatophyte infections and asymptomatic carriage. Therefore, all veterinary activities with wild animals should be performed using protective gloves and continuous environmental disinfection to avoid contagion [21, 22]. In the natural environment, the risk of transmission of infection from hedgehogs is lower, and more attention should be paid to soil contamination, as it can indirectly pose a risk of infection to humans and other animals, including pets. It is commonly known that some zoophilic dermatophytes also have soil reservoirs [24].

The isolation and identification of the typically geophilic dermatophyte *Nannizzia gypsea*, as well as a species of dual zoophilic and geophilic nature, i.e. *Nannizzia nana*, from free-living hedgehogs confirms that the reservoirs of dermatophytes can change and the soil is a favourable environment for these pathogens. It should be noted that *Nannizzia fulva* was isolated for the first time from hedgehogs in our study. This geophilic dermatophyte species was detected in a variety of soil sources, especially shady and wet places rich in organic substances, e.g. gardens, public grassland, sludge, and locations full of animal keratin materials in Iran [43, 44], Tunisia [45], India [46], Saudi Arabia [47], USA [48], and Brazil [49]. Additionally, *N. fulva* was more often isolated from animal habitats in parks and city gardens than *N. gypsea*, which is typically related to soils in home range areas [50]. Hence, hedgehogs found in urban regions may be infected. The presence of geophilic dermatophytes *N. gypsea* in asymptomatic carrier status animals was reported as well. These results are consistent with those published by Le Barzic et al. [12] based on research carried out in France at a wildlife rescue centre. This may indicate that hedgehogs are an important carrier of geophilic dermatophytes, regardless of the geographic region. Nonetheless, the hedgehog-specific fungus *T. erinacei* was the most frequent dermatophyte species observed (29.9%) in both symptomatic and asymptomatic animals. The host range of this species seems to be narrow and identification thereof in other animals and in human cases of infection is rare compared to that in hedgehogs [51–54]. Contrarily, *T. mentagrophytes* found in 17.9% of cases in hedgehogs with dermatophytosis and asymptomatic carriers have been reported from a broad but poorly known spectrum of domestic animals and human zoophilic origin mycoses [1, 2, 55, 56]. This species of dermatophyte has already been isolated from hedgehogs by Le Barzic et al. [12] in a monitoring study conducted in wildlife rehabilitation centres. Additionally, these authors indicate that *T. mentagrophytes* morphology is relatively similar to that of *T. erinacei*, which may cause confusion in identification analyses, especially samples from wild animals. In part, this is related to the extensive taxonomic rearrangements in the dermatophyte group since 2017 [5, 57]. Therefore, identification by molecular techniques should always be performed, as some isolates do not develop characteristic features to ensure the credibility of the morphological examination alone.

In the context of the isolation of *T. mentagrophytes* from hedgehogs, the detection of terbinafine-resistant strains is clinically important. Allylamine resistance has already been reported in Asian and European countries [2, 3, 32, 58–60]. Moreover, the prevalence of terbinafine-resistant clinical isolates of *T. mentagrophytes* ranged from less than 1 in Switzerland [32] to more than 70% in India [61]. Current scientific reports do not clearly explain the cause of this phenomenon. Terbinafine resistance can be selected after treatment in the patient or can be innate [2, 62]. Our results are not able to indicate clearly the mechanism that might exert an effect on resistance in free-living hedgehogs. Nevertheless, in two cases, in vitro resistance to terbinafine (MIC = 2 µg/ml) was demonstrated for *T. mentagrophytes* isolates obtained from animals with dermatophytosis. In addition, the Leu393Phe amino acid substitution in the squalene epoxidase protein was identified as responsible for the resistance. This substitution, together with Phe397Leu, was reported in earlier studies conducted on terbinafine-resistant *Trichophyton* spp. isolates [2, 62–65]. Furthermore, Gln408Leu, Leu393Ser, Leu398Phe, Phe402Leu, and other less common substitutions were also correlated with high MIC values of terbinafine in dermatophytes and yeast-like fungi [58, 66]. Many researchers report that terbinafine resistance is also geographically limited and regional predispositions to differential sensitivity to antifungal drugs are noted [2, 67]. Based on the literature, it can be concluded that the greatest prevalence of resistant dermatophytes occurs in Asian countries, especially India. Nevertheless, European countries, including Poland [1, 2], Finland [59], Denmark [68], Switzerland [58], and Russia [60], are not free from dermatophyte isolates that are resistant to conventional treatment. Our study shows that hedgehogs may be a poorly studied reservoir of these strains. Moreover, the infectious agent of dermatophytoses in the free-living hedgehogs described here may have originated also from the soil, i.e. a non‐animal environment that can be an additional reservoir of *T. mentagrophytes*. Therefore, the detection of drug-resistant strains, animal species affected, and disease state and the indication of their geographical range is an important task for medical mycologists.

A strikingly high incidence of zoonotic *T. benhamiae* infections is currently reported in various European countries [69]. The prevalence of this pathogen isolated from guinea pig breeds and pet shops reaches up to 90% [70]. However, this dermatophyte has been also reported in dogs, rabbits, cats, North American porcupines, alpacas, foxes, and various small rodents [27, 71]. So far, *T. benhamiae* has not been reported in hedgehogs, either free-living or kept as pets, or any other wild animals. Probably due to the presence of this species of dermatophyte in rodents, whose feeding niches may be similar to those of urban hedgehogs, mechanical transmissions are possible. Thus, it is possible that the host range for *T. benhamiae* is wide, and subsequent monitoring studies will reveal new animal reservoirs. Moreover, the isolation of *M. canis* from urban hedgehogs seems interesting as well. This dermatophyte is usually correlated with a domestic environment and is associated with dogs and cats [72]. Nonetheless, in the last year, *M. canis* was isolated from dermatological lesions of Eastern cottontail (*Sylvilagus floridanus*), which is a synanthropic animal easily observed near urban centres with high population density. The fungus was also isolated from hair and soil samples collected in front of burrow entrances of alpine marmot (*Marmota marmota*) [73, 74]. This may indicate potential *M. canis* reservoirs in wild animals, especially those that forage in urban areas.

Recently, the literature has presented a suggestion that the molecular approach for the identification of dermatophytes in samples is trustworthy, rapid, and significantly more reliable than conventional diagnostic methods [28]. The real‐time PCR techniques are increasingly frequently used in the detection of dermatophytes due to their high specificity and sensitivity, even in cases with negative cultures [1, 27, 28]. Our studies have proved that the use of the real-time PCR technique with pan-dermatophyte primers detects the presence of dermatophytes in the sample with a 35.3% (100% vs. 64.7%) and 26.4% (45.3% vs. 18.9%) higher efficiency than cultures in the analysis of symptomatic and asymptomatic hedgehogs, respectively. Nevertheless, talking about the status of a carrier, it is necessary to indicate the presence of living infectious elements of dermatophytes [1, 24]. These can only be demonstrated using conventional methods with obtaining cultures [28]. Therefore, the molecular methods for identifying dermatophytes cannot be used alone, without reference to obtaining culture by conventional methods.

In conclusion, the high prevalence of symptomatic infections and asymptomatic carriers in free-living hedgehogs detected in this study stresses the risk of dermatophyte dissemination in the natural environment and indicates possible zoonotic transmissions. The identification of geophilic dermatophyte species may also indicate the currently changing ecological niches of these fungi. Moreover, we have revealed that in vitro terbinafine resistance may emerge with the analysed mutations in the squalene epoxidase gene in wildlife animals. Finally, further research is needed to elucidate whether the asymptomatic carriage is related only to mechanical transport of dermatophytes or infection with low-virulence isolates as well.

## Data Availability

The datasets generated during the current study are available from the corresponding author on reasonable request.
